# Properties of viable lyopreserved amnion are equivalent to viable cryopreserved amnion with the convenience of ambient storage

**DOI:** 10.1371/journal.pone.0204060

**Published:** 2018-10-02

**Authors:** Sandeep Dhall, Malathi Sathyamoorthy, Jin-Qiang Kuang, Tyler Hoffman, Matthew Moorman, Anne Lerch, Vimal Jacob, Steven Michael Sinclair, Alla Danilkovitch

**Affiliations:** Osiris Therapeutics Inc., Columbia, MD, United States of America; Cedars-Sinai Medical Center, UNITED STATES

## Abstract

Human amniotic membrane (AM) has a long history of clinical use for wound treatment. AM serves as a wound protective barrier maintaining proper moisture. AM is anti-inflammatory, anti-microbial and antifibrotic, and supports angiogenesis, granulation tissue formation and wound re-epithelialization. These properties of AM are attributed to its native extracellular matrix, growth factors, and endogenous cells including mesenchymal stem cells. Advances in tissue preservation have helped to overcome the short shelf life of fresh AM and led to the development of AM products for clinical use. Viable cryopreserved amnion (VCAM), which retains all native components of fresh AM, has shown positive outcomes in clinical trials for wound management. However, cryopreservation requires ultra-low temperature storage and shipment that limits widespread use of VCAM. We have developed a lyopreservation technique to allow for ambient storage of living tissues. Here, we compared the structural, molecular, and functional properties of a viable lyopreserved human amniotic membrane (VLAM) with properties of VCAM using *in vitro* and *in vivo* wound models. We found that the structure, growth factors, and cell viability of VLAM is similar to that of VCAM and fresh AM. Both, VCAM and VLAM inhibited TNF-α secretion and upregulated VEGF expression *in vitro* under conditions designed to mimic inflammation and hypoxia in a wound microenvironment, and resulted in wound closure in a diabetic mouse chronic wound model. Taken together, these data demonstrate that VLAM structural and functional properties are equivalent to VCAM but without the constraints of ultra-low temperature storage.

## Introduction

Human placental tissue, particularly the amniotic membrane (AM), has a long history of use as a biological dressing for acute and chronic wounds [1–4]. AM is anti-inflammatory, antimicrobial, and antifibrotic [5–7]. In addition, AM maintains a moist environment in the wound, supports angiogenesis, granulation of the wound bed, and wound epithelialization [3,8]. These functional properties are attributed to the composition of tissue, including a collagen-rich structural extracellular matrix, growth factors and cytokines, and endogenous viable epithelial cells, fibroblasts, and mesenchymal stem cells [1,2,9–11]. However, due to restricted availability and short shelf life of fresh tissue, use of fresh AM is limited. Advances in tissue preservation led to the commercialization of AM. The goal of preservation is to retain all components of fresh tissue in the native state for extended periods of time that are sufficient to complete donor and tissue testing, and make “point of care” tissue products available on demand. While various preservation methods have been developed for placental tissue processing, most of these methods destroy native viable cells. Cryopreservation, a common method for long-term storage of viable cells and tissues, involves the cooling of samples to very low temperatures in the presence of cryoprotective agents followed by storage at ultra-low temperatures [12]. However, tissue thickness, the presence of multiple structural layers and various types of cells makes sample preservation challenging. Previous studies attempted to preserve viable cells within the AM reported varying results [13–15]. Only recently has a cryopreservation method been developed to allow storage of AM for prolonged periods without compromising cell viability post-thaw [16–18].

Studies show that preservation of AM components, including ECM, growth factors, and viable cells, is required for the retention of full spectrum biological activity of the tissue [18]. Viable cryopreserved AM (VCAM) retains ECM, growth factors, and viable cells of fresh tissue [18] and demonstrates a higher magnitude of anti-inflammatory, antioxidant, angiogenic, and chemoattractive activities as compared to devitalized cryopreserved AM [17,19,20]. The use of VCAM as an adjunct to standard wound care for chronic wounds shows better clinical outcomes in comparison to standard of care alone [21]. In a multicenter, randomized, controlled clinical trial, VCAM application to chronic diabetic foot ulcers resulted in a significantly higher proportion of closed wounds, with faster wound closures and fewer wound-related adverse events [21,22]. Other VCAM retrospective and prospective clinical studies reported positive clinical outcomes in patients with acute and chronic wounds of various etiologies, and in a variety of surgical procedures [22–25]. Comparative effectiveness studies of VCAM versus a devitalized dehydrated amnion chorion product showed that the use of VCAM resulted in higher wound closure rate of larger, more difficult to treat wounds [26,27]. Clinical results are consistent with results from *in vitro* studies providing further support for preserving all components of native placental tissues.

A caveat of cryopreservation is the requirement for the “cold chain” storage and distribution of cryopreserved products, thus significantly limiting the use of VCAM. The requirement of specialized equipment to maintain ultra-low temperatures during preservation, storage, and shipment is a major drawback of cryopreservation [28]. This limitation complicates distribution logistics and is associated with high cost, thus limiting widespread clinical use of products containing living cells. Therefore, the development of a preservation technology for living cells and tissues to allow for long-term ambient storage is important for the advancement of cellular therapies.

The existence of desiccation-tolerant animals, or anhydrobiotes, suggests that dehydration of mammalian cells and tissues can be performed without compromising viability [29]. Some of the biochemical changes associated with anhydrobiote dehydration have been documented [30] and can provide strategies for the preservation of mammalian cells and tissues. The sugar trehalose was found to protect the intracellular organelles of anhydrobiotic animals exposed to desiccation [31]. Researchers have experimented with trehalose and other sugars for cell preservation and demonstrated that normal function is retained after drying and rehydration of platelets [32]. Based on accumulated data on cell preservative agents, lyophilization processes, and preliminary protocols for mammalian cell drying [12,31,33–39], we developed a lyopreservation technique for the preservation of living tissues allowing for storage at ambient temperature.

The purpose of the present study was to evaluate the structural and functional properties of human AM preserved by the lyopreservation technique and to compare its properties to those of VCAM. We show that following rehydration, tissue structure and cell viability of fresh AM are maintained in both cryopreserved and lyopreserved AM. More importantly, lyopreserved AM (VLAM) retains functionality as demonstrated in both *in vitro* and *in vivo* models. In summary, while both cryopreservation and lyopreservation methods yield viable functional amniotic tissue, the latter allows for storage at ambient temperatures, eliminating the requirement of ultra-low temperature equipment and making cellular and tissue products more accessible for widespread use.

## Materials and methods

### Tissue procurement and ethics statement

Commercially available human term placentas were provided by The National Disease Research Interchange (Philadelphia, PA) and Cord Blood America, Inc. (Las Vegas, NV). Tissue procurement and ethics statement were provided by The National Disease Research Interchange (Philadelphia, PA), Cord Blood America, Inc. (Las Vegas, NV). Tissues were collected from eligible donors after obtaining written, informed consent.

### Isolation of amnion from human placenta

Placental tissue processing from human normal full-term placentas was carried out as previously described [17]. Briefly, AM was separated from the umbilical cord and chorion, cleaned, and treated with an antibiotic cocktail [17]. Residual antibiotics was removed with a Dulbecco's Modified Eagle Medium (DMEM; GE Healthcare, Logan, UT) wash, and AM was cut into 5x5 cm^2^ (25 cm^2^) pieces. Viable cryopreserved AM (VCAM) and viable lyopreserved AM (VLAM) were prepared using the cryopreservation and lyopreservation protocols described below. For the *in vitro* inflammatory assay and viability staining experiments, 3x4 cm^2^ (12 cm^2^) rectangular and 16 mm (~2 cm^2^) diameter disc samples were cut out of the 25 cm^2^ pieces of VLAM and VCAM post-rehydration or post-thaw, respectively. Fresh, VLAM, and VCAM samples for each experiment were prepared from the placentas derived from the same donors. Tissue processing was performed with aseptic technique using sterile reagents and instruments in a biosafety cabinet. To retain viable cells, neither lyophilized nor cryopreserved amnions were terminally sterilized by radiation or by any other methods. The asepsis of the process was confirmed by USP <71> sterility tests.

### Tissue preservation

#### Cryopreservation

Human amnion samples (25 cm^2^) were mounted on nitrocellulose paper (GE Lifesciences, Pittsburgh, PA) and placed in FP-90 cryobags (Charter medical, Winston-Salem, NC) containing a chilled 5% DMSO (Sigma, St. Louis, MO) and 1% human serum albumin (Octapharma, Hoboken, NJ) in saline solution. Samples were incubated at 4^o^ C for 30 min, and then placed into a pre-chilled styrofoam container and transferred to a -80^o^ C freezer for a controlled-rate freezing at 0.4° C/minute cooling established with a temperature probe. All samples were stored at a -80^0^ C prior to experiments.

#### Lyopreservation

Tissue samples were saturated in a 0.25M trehalose (Avantor, Center Valley, PA) in saline solution at room temperature for a minimum of 30 min in a volume sufficient to submerge tissues. To ensure sterility during lyophilization, pieces of tissue were packaged in two sterile Tyvek pouches (Rollprint, Addison, IL) prior to being loaded in a lyophilizer. The packaged samples were then placed in a lyophilizer (Lyostar II, SP Scientific, Warminster, PA). Freezing was performed for 120 min after cooling to -50^o^ C at 1.16° C/min. Primary drying was performed by lowering the chamber pressure to 200 mTorr at -20^o^ C and for 360 min. Primary drying was immediately followed by a three-phase secondary drying. In the first phase, the vacuum pressure was held at 200 mTorr and the shelf temperature was raised to 0^o^ C at a rate of 3^o^ C/min and held for 360 min. In the second phase, the vacuum pressure was held at 200 mTorr and the temperature was raised to 20^o^ C at a rate of 3^o^ C/min and held for 360 min. In the third and final phase, the vacuum pressure was held at 200 mTorr and temperature was raised to 30^o^ C and held for 2,760 min.

### VLAM rehydration procedure

VLAM was rehydrated by submerging the lyopreserved membrane in sterile PBS for 30 min at room temperature.

### VCAM thawing procedure

VCAM was thawed by placing the frozen bag in a water bath at 37^o^ C for 5–15 min. To avoid a DMSO toxic effect on viable cells immediately after thawing, VCAM was transferred from the bag with cryosolution into a basin with sterile PBS.

### Detection of growth factors and cytokines in AM samples

Placentas from six donors were used for evaluation of growth factor and cytokine profiles. 25 cm^2^ samples of fresh AM, VCAM and VLAM were prepared from the placentas derived from the same donors. VLAM was rehydrated using sterile PBS, and VCAM was thawed prior to experiments as described under “VLAM rehydration procedure” and “VCAM thawing procedure”, respectively. Tissue extracts were prepared by homogenizing fresh AM, VCAM, or VLAM in a T-PER buffer (Thermo Fisher Scientific, Waltham, MA) supplemented with a protease inhibitor cocktail (Sigma, location) using a gentleMACS dissociator (Miltenyi Biotec, Auburn, CA). Tissue extracts were clarified by centrifugation (Eppendorf, Hauppauge, NY) at 14,000 rpm for 15 min. Clarified tissue extracts were analyzed using a multiplex growth factor panel kit (LXSAHM-15, R&D Systems) and Bio-Plex MAGPIX (Bio-Rad, Hercules, CA). The test panel included Interleukin 10 (IL-10), Interleukin-1 receptor antagonist (IL-1RA), Platelet-derived growth factor BB (PDGF-BB), Basic fibroblast growth factor (bFGF), Stromal cell-derived factor 1 alpha (SDF-1α), and Angiogenin 1 (Ang-1).

### Cell viability assays

#### LIVE/DEAD fluorescent tissue staining

16 mm diameter discs were cut from 25 cm^2^ samples of fresh AM, VCAM and VLAM and used for staining. VLAM was rehydrated using sterile PBS. VCAM was thawed prior to staining, detached from the nitrocellulose backing using forceps and washed in PBS for 1 min before transferring into the staining solution. Samples were stained with a LIVE/DEAD Viability/Cytotoxicity Kit (Thermo Fisher Scientific, Waltham, MA) following the manufacturer’s instructions and imaged using the EVOS™ FL Auto Imaging System and Celleste™ Image Analysis Software (Thermo Fisher Scientific, Waltham, MA). Cell viability was quantified using acquired tissue images with the Smart Segmentation and Count feature of Celleste Image Analysis Software. After counting total number of cells, viable cells, and dead cells, the percentage of viable cells was calculated as: [number of viable cells/number of total cells]x100%. At least 50 images per disc for each donor were acquired and evaluated for these calculations.

### *In vitro* testing

#### Anti-inflammatory activity

Anti-inflammatory activity of VLAM and VCAM was evaluated by inhibition of tumor necrosis factor α (TNF-α) release from activated human peripheral blood mononuclear cells (PBMCs). A vial of cryopreserved PBMCs (Precision for Medicine, Bethesda, MD) was thawed, and cells were resuspended in DMEM+10% FBS at a concentration of 1x10^6^ cells/ml. PBMCs were stimulated with Cluster of Differentiation (CD) CD3/CD28 antibodies (BD Biosciences, San Jose, CA) at 10 ng/ml and incubated for 48 h in the presence of a 12 cm^2^ of VCAM or VLAM, which were cut with a scalpel into 6–8 smaller pieces prior to placing in a 24-well plate at 37° C and 5% CO_2_. Stimulated and unstimulated PBMCs alone served as positive and negative controls, respectively. After 48 h, culture supernatants from each well were collected and centrifuged at 14,000 rpm for 5 min. TNF-α levels in each supernatant were quantified using a Bioplex human cytokine assay (BioRad, Hercules, CA) according to the manufacturer’s protocol.

#### Effect of *in vitro* chronic wound environment on vascular endothelial growth factor (VEGF) secretion

25 cm^2^ samples of VLAM or VCAM were cut with a scalpel into 10–12 small pieces, all of which were placed into wells of a 24-well cell culture plate with DMEM containing 10% FBS. VLAM and VCAM were incubated under conditions mimicking a chronic wound microenvironment. This microenvironment was simulated by adding 100 ng/ml bacterial lipopolysaccharide (LPS) (Sigma, St. Louis, MO) and 10 ng/ml TNF-α (R&D Systems, Minneapolis, MN) to the culture medium, and incubating the plates in a hypoxic chamber (2% O_2_). A parallel experiment carried out under normoxic (21% O_2_) conditions lacking LPS and TNF-α served as a baseline. After 4 days, membranes were snap-frozen in liquid nitrogen, and tissues were homogenized in T-PER (Thermo Fisher Scientific, Waltham, MA) lysis buffer supplemented with a protease inhibitor cocktail (Sigma, St. Louis, MO) and 8 mM guanidine hydrochloride (Sigma, St. Louis, MO). VEGF levels in tissue lysates were quantified using a VEGF Quantikine ELISA kit (R&D Systems, Minneapolis, MN) according to the manufacturer’s recommendations.

### *In vivo* testing

#### Animals

Diabetic (db/db) mice (Jackson Laboratories, Bar Harbor, ME) were used in these studies. Six-month old animals were housed at the Sobran BioScience vivarium at Johns Hopkins University. All experimental protocols were approved by the Sobran’s Institutional Animal Care and Use Committee. All methods were performed in accordance to the guidelines and regulations of The Association for Assessment and Accreditation of Laboratory Animal Care International. All mice were fed a standard chow diet. Mice were individually housed in cages with exterior dimensions 14.7"L x 9.2"W x 5.5"H (Innovive Caging, San Diego, CA) and kept on 12 / 12 light dark cycle in a clean vivarium environment.

#### Chronic db/db wound model

Chronic wounds were developed as previously described [40]. Briefly, 20 min prior to creating 7 mm wounds, mice were treated once intraperitoneally with 3-amino-1,2,4-triazole (Aldrich Chemistry, St. Louis, MO), a catalase inhibitor, at 1 g/kg body weight. Immediately post injury, wounds were treated topically with mercaptosuccinic acid (MSA) (Sigma, St. Louis, MO), an inhibitor of glutathione peroxidase, at 150 mg/kg body weight and covered with Tegaderm™ (3M, St. Paul, MN). Prior to the wounding, the animals were anesthetized using 1% isoflurane plus oxygen inhalation. Immediately following surgery and prior to their recovery from anesthesia, analgesic substances (buprenorphine, 0.05 mg/kg, SQ) were administered. In case of stress (noticeable lethargy (lack of movement during ~ 5 min of observation), hunched posture for >5 min, ignorance of ruffled/poorly groomed fur for ~ 15 min, slow shallow and labored breathing for >5 min), the animal was placed on a heating pad for 30 mins to avoid post-surgical hypothermia. A second dose (0.05 mg/kg, SQ) was administered 6 to 12 h post-recovery. Within 20 days, wounds became chronic. These chronic wounds were treated weekly with topical applications of either normgel, VLAM, or VCAM. Each experimental group was comprised of seven animals per group. Animal weights and wound photos were collected weekly. Wound photos were used for calculation of the wound area. Animals were euthanized by CO_2_ inhalation. Tissue samples were collected following wound closure and processed for histological evaluation.

### Histological and fluorescent immunohistochemical staining procedures

#### Analysis of AM samples

VLAM (post-rehydration), VCAM (post-thaw), and fresh AM were fixed in 4% paraformaldehyde and processed for paraffin embedding. Tissue sectioning and staining were performed by Histoserv Inc. (Germantown, MD) using standard protocols for hematoxylin and eosin (H&E) and Masson’s trichrome (MT) staining.

#### Wound sample analysis

Wound tissue samples were fixed in 4% paraformaldehyde and processed for Optimal Cutting Temperature compound embedding [40] (Sakura Finetek, Torrance, CA) for sectioning. For detection of α smooth muscle antigen (αSMA), rabbit αSMA polyclonal antibodies (Abcam #5694, Cambridge, MA) were used followed by goat anti-rabbit AlexaFlour 568-labeled IgG (ThermoFisher #A-11011, Rockford, IL). For detection of blood vessel endothelial cell marker CD31, a rat CD31 monoclonal antibody (Abcam #7388, Cambridge, MA Cambridge, MA) was used followed by goat anti-rat AlexaFlour 568-labeled IgG (ThermoFisher #A-11077, Rockford, IL). For detection of Collagen Type IV, we used Collagen IV rabbit polyclonal antibodies followed by goat anti-rabbit AlexaFlour 568-labeled IgG (ThermoFisher #A-11011, Rockford, IL). Negative controls were performed by staining sections with goat anti-rat AlexaFlour 568-labeled IgG (ThermoFisher #A-11077, Rockford, IL) or goat anti-rabbit AlexaFlour 568-labeled IgG (ThermoFisher #A-11011, Rockford, IL). Cell nuclei were stained with 4',6-diamidino-2-phenylindole (DAPI) in Vectashield (Vector Laboratories, Burlingame, CA) mounting medium.

### Statistical analysis

Results are presented as mean ± SD. Student's T-test was used to determine the significance of differences between groups, whereby p<0.05 was considered significant.

## Results

### Native tissue matrix, growth factors and endogenous viable cells are preserved in VLAM

**Fig 1:**
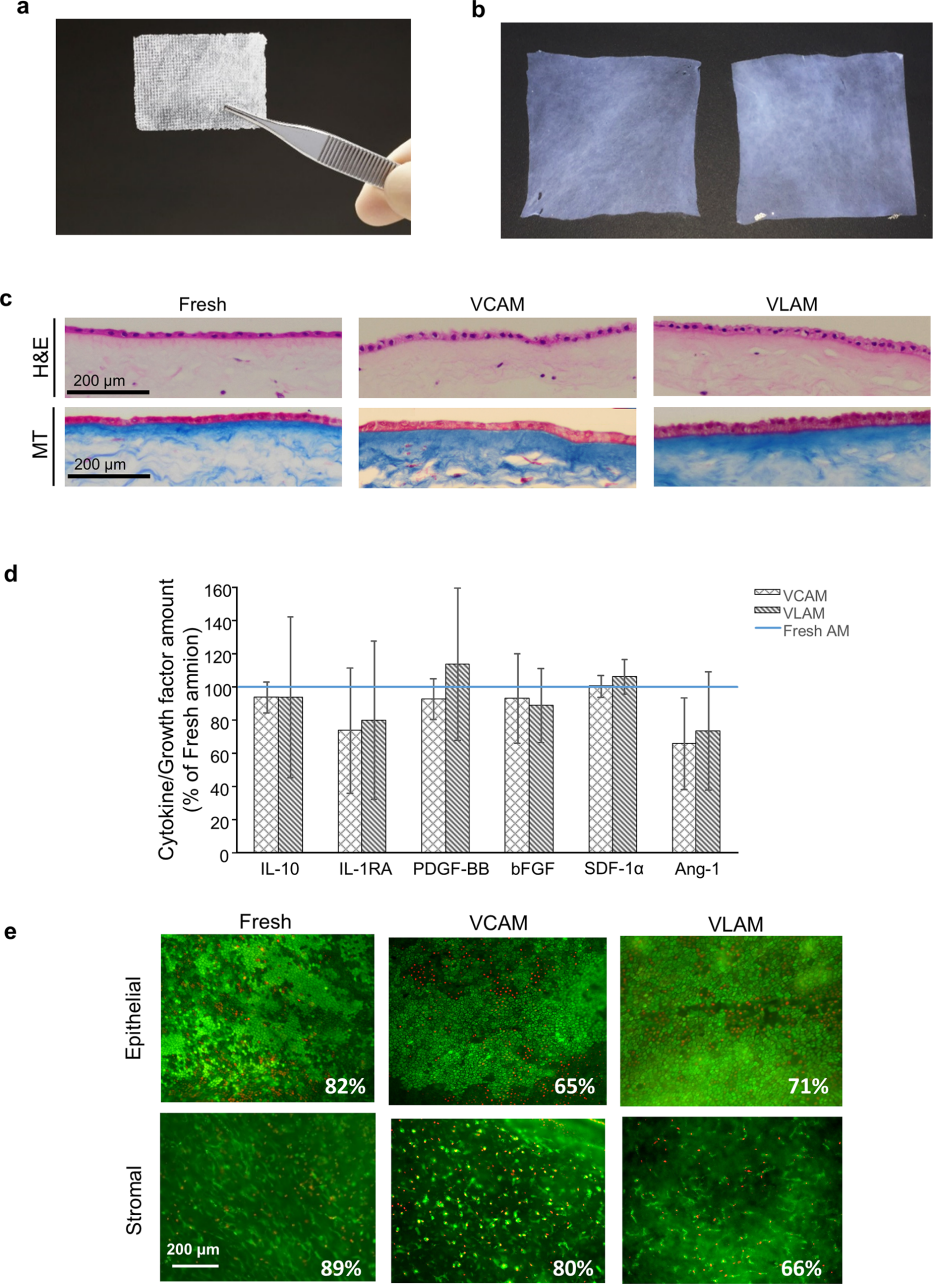
VCAM and VLAM retain integrity of fresh AM. (a) Visual appearance of VLAM. (b) Visual appearance of VCAM post-thaw (on the left) and VLAM post-rehydration (on the right). (c) Histological images of fresh AM (left panels), VCAM (middle panels) and VLAM (right panels): Hematoxylin and eosin (H&E) **(**top panels**)** and Masson’s trichrome (MT) (bottom panels) staining. (d) Presence of interleukin-10 (IL-10), interleukin-1 receptor antagonist (IL-1RA), platelet derived growth factor BB (PDGF-BB), basic fibroblast growth factor (bFGF), stem cell derived factor-1α (SDF-1α) and angiopoietin-1 (Ang1) in VCAM and VLAM. Average growth factor levels for 6 donors are shown relative to fresh AM. (e) Cell viability assessment by LIVE/DEAD staining of fresh AM (left panels), VCAM (middle panels) and VLAM (right panels) derived from the same placental donor. Representative images show viable cells (green) and dead cells (red) in epithelial (top panels) and stromal (bottom panels) layers in fresh AM, VCAM and VLAM. Percentage of viable cells is indicated for each image.

Visually, VLAM appears as a thin off-white-colored graft (Fig 1A). However, after rehydration, VLAM is indistinguishable from VCAM post-thaw (Fig 1B) and from fresh AM (data not shown). Blinded investigators were unable to distinguish VLAM from VCAM or from fresh samples by visual assessment or by manual stretching as a measure of mechanical strength of the tissues.

Histological analysis of VLAM was performed to evaluate structural integrity of the tissue (i.e. extracellular matrix or ECM) after lyophilization and compared to that of fresh AM and VCAM. Tissue sections were stained with hematoxylin and eosin (H&E) to assess tissue morphology and with Masson’s trichrome (MT) to visualize collagen content and distribution within the tissue (Fig 1C). H&E staining (Fig 1C, top panels) showed the presence of a single layer of epithelial cells attached to a basement membrane, followed by a compact layer, and a stromal layer containing sparse cells with fibroblastic morphology in AM samples. The structural matrix in VLAM (Fig 1C, right top panel) is comparable to those of fresh AM and VCAM (Fig 1C, left and middle top panels, respectively). MT staining (Fig 1C, bottom panels) revealed a similar distribution of collagen in VLAM as compared to fresh AM and VCAM.

A broad variety of growth factors critical for wound healing are present in AM tissue [10,41]. We tested fresh, VCAM, and VLAM tissue extracts for the presence of six factors important for tissue repair and regeneration. Data confirmed that both VCAM and VLAM retain growth factors that are present in fresh AM. The graph shows mean levels of six growth factors in VCAM and VLAM for six donors relative to fresh AM (Fig 1D): interleukin-10 (IL-10), interleukin-1 receptor antagonist (IL-1RA), platelet derived growth factor BB (PDGF-BB), basic fibroblast growth factor (bFGF), stromal cell-derived factor-1α (SDF-1 α) and angiopoietin-1 (Ang1) (Fig 1D). There were no significant differences in growth factor levels between fresh AM, VCAM, and VLAM.

The presence of viable cells was confirmed in both epithelial and stromal layers of fresh AM, VCAM and VLAM using the LIVE/DEAD viability/cytotoxicity assay. Visually the majority of cells in both epithelial (Fig 1E, top panels) and stromal (Fig 1E, bottom panels) layers of fresh AM, VCAM and VLAM were viable. For calculation of average % cell viability, a total of 1000 images from 50 donors was analyzed. The storage time prior to analysis ranged from three weeks to six months. On average, 70%±9% of viable cells were detected in VLAM, as compared to 86%±11% in fresh AM and 73%±7% in VCAM. There were no significant differences between the number and distribution of viable cells across the tissues between fresh AM (Fig 1E, left panels), VCAM (Fig 1E, middle panels) and VLAM (Fig 1E, right panels). Also, cells in fresh AM, VCAM and VLAM remained viable for at least for three weeks when samples were placed in tissue culture medium (data not shown). Taken together, these data indicate that similar to VCAM, VLAM retains ECM architecture, growth factors, and endogenous viable cells present in fresh AM.

### VLAM anti-inflammatory and pro-angiogenic activities *in vitro*

**Fig 2:**
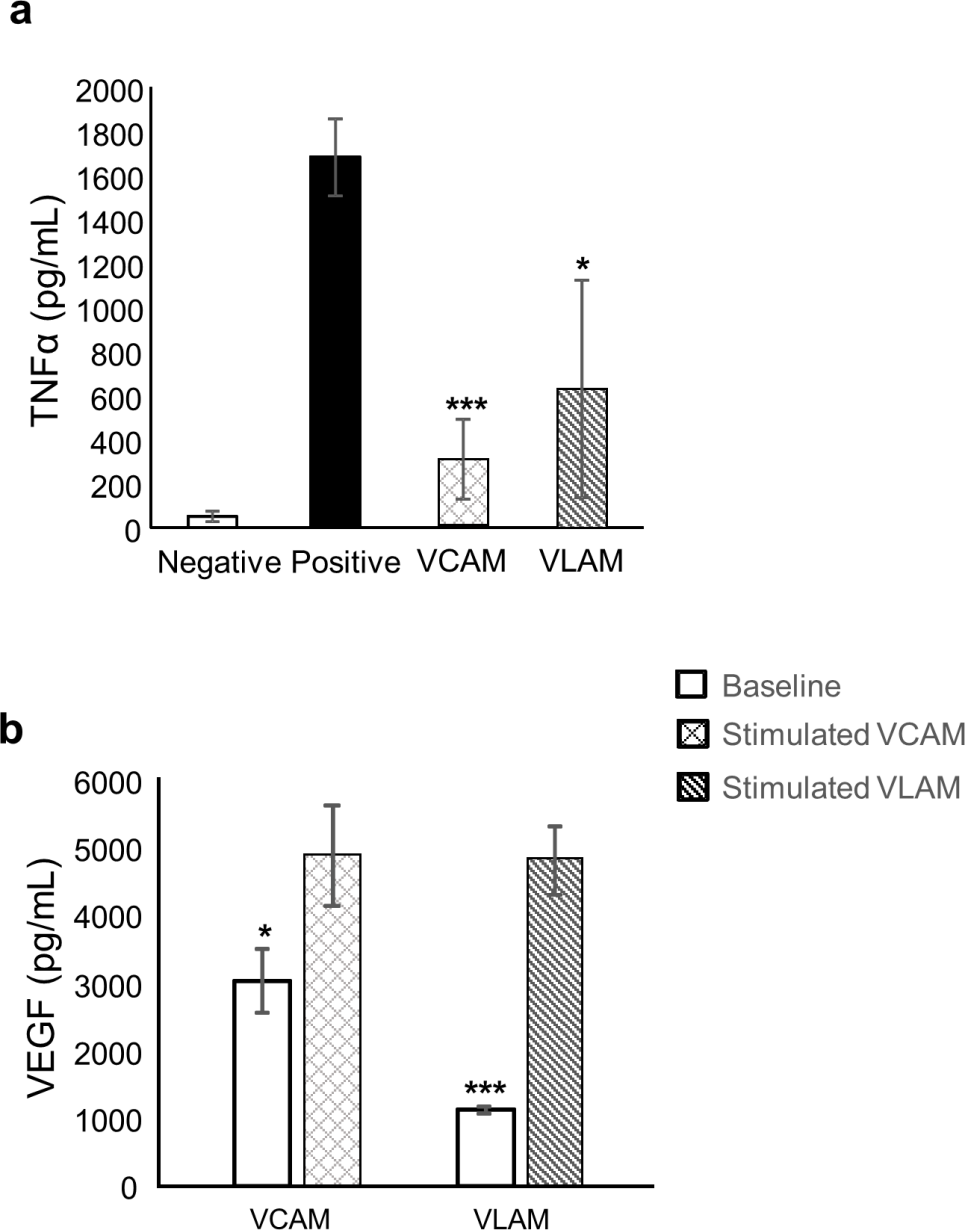
VLAM anti-inflammatory and pro-angiogenic activities *in vitro*. (a) Anti-inflammatory activity of VLAM was evaluated by inhibition of TNF-α release from stimulated human peripheral blood mononuclear cells (PBMCs). Unstimulated and stimulated PBMCs alone served as positive and negative controls, respectively. *p <0.05, ***p<0.001 compared to the stimulated PBMC control. (b) Pro-angiogenic activity of VLAM was determined by VEGF expression in a chronic wound microenvironment (see Methods). Baseline represents data obtained under normoxic conditions (see Methods). Stimulated VLAM and VCAM are tissues exposed to 100 ng/ml bacterial LPS, 10 ng/ml TNF-α in a hypoxic chamber (2% O_2_). VCAM shown for comparison. Values represent the average of three independent experiments. *p <0.05, ***p<0.001 compared to control.

AM has anti-inflammatory and pro-angiogenic properties, both of which are beneficial for tissue repair [9,10,42]. Previously we have reported that VCAM retains anti-inflammatory and pro-angiogenic activities of AM [17,20]. Here, we assayed the anti-inflammatory activity of VLAM by measuring its effect on TNF-α secretion by CD3/CD28 antibody-activated peripheral blood mononuclear cells (PBMCs) (Fig 2A). TNF-α secretion by activated PBMCs was markedly reduced in the presence of VLAM and VCAM, which served as a positive control for this experiment (Fig 2A). This data indicates that VLAM retains anti-inflammatory activity of AM. Next, we investigated the pro-angiogenic potential of VLAM *in vitro*. We found that hypoxia, LPS and TNF-α, which were used to mimic a wound environment, upregulated VEGF expression in VLAM and VCAM (Fig 2B). Taken together, these data demonstrate that similar to VCAM, VLAM retains anti-inflammatory and pro-angiogenic properties of AM.

### VLAM accelerates wound closure in a diabetic mouse chronic wound model

**Fig 3:**
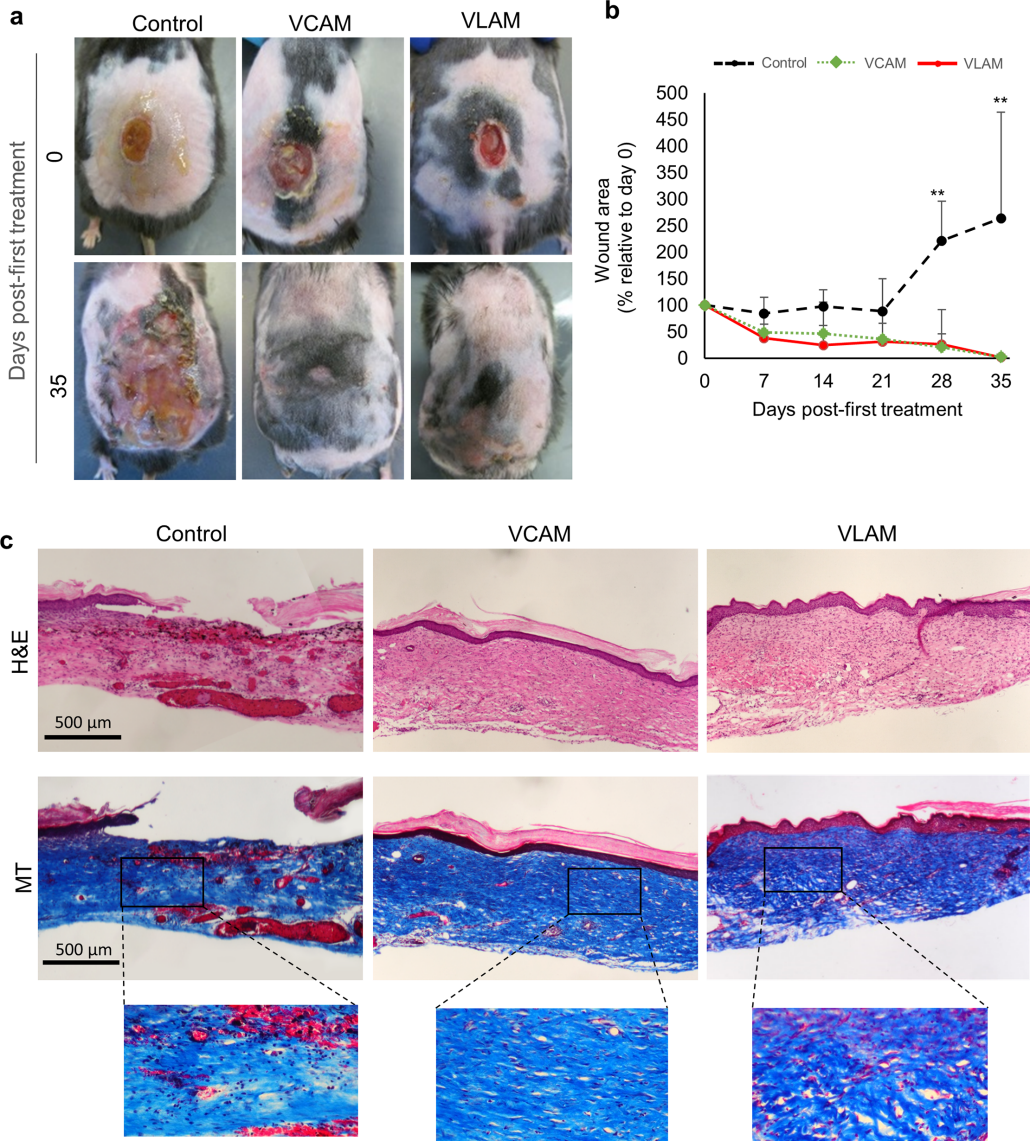
Effects of VLAM in a diabetic mouse chronic wound model. (a) Representative images of wounds at day 0 and day 35 following treatment. (b) Wound area measured weekly and expressed as a percent of wound area at day 0. Data represent the average of 7 mice per treatment group. **p<0.01 compared to control. (c) Histological analysis of wound tissue using H&E **(**top panels**)** and MT staining **(**bottom panels**)** at day 35 (VCAM and VLAM, middle and right panels, respectively) or day 94 (control). A saline gel-treated group served as the negative control (left panels). MT images were captured at 4X and 20X.

We used a db/db mouse chronic wound model to evaluate the effect of VLAM applications on wound healing and to compare with application of VCAM. VLAM or VCAM were applied weekly to chronic wounds in mice until closure was achieved in one of the treatment groups. For this experiment, saline gel was used as a negative control. Both VLAM and VCAM application resulted in wound closure after 35 days of treatment (Fig 3A and Figure a in S1 Fig). In contrast, wounds in control mice (saline gel treated) remained open for longer than 90 days (data not shown), consistent with previously reported results [40]. Wound size (Fig 3B) and animal weights (Figure b in S1 Fig) were measured weekly throughout the course of treatment. Histological analysis of wound samples from VLAM- and VCAM-treated wounds at day 35 and from control wounds at day 73 (from treatment initiation) was performed to assess the quality of repaired tissue. H&E staining of tissue from VCAM- and VLAM-treated wounds revealed proper granulation and re-epithelialization consistent with a normal mouse dermis and epidermis (Fig 3C, top middle and right panels). In contrast, wounds in the control group were not re-epithelialized (Fig 3C, top left panel). MT staining showed proper collagen deposition and the presence of “basket weave”-like structures representing mature collagen in both VCAM- and VLAM-treated wounds (Fig 3C, bottom middle and right panels). No evidence of “basket weave”-like structures was observed in control wounds (Fig 3C, bottom left panel).

### VLAM promotes vascularization and basement membrane formation in diabetic mouse chronic wounds

**Fig 4:**
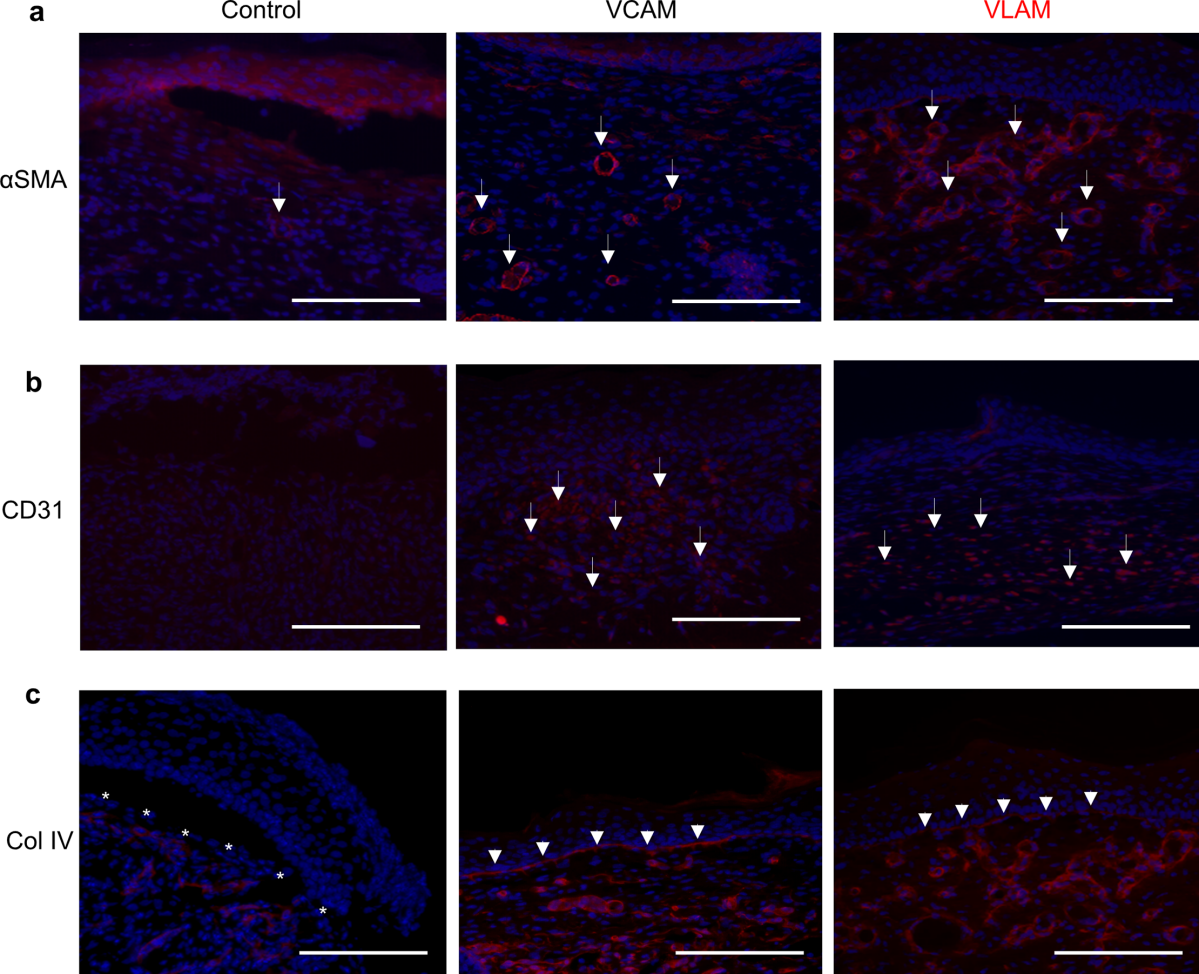
Neovascularization and basement membrane formation in VCAM- and VLAM-treated wounds. Effect of VCAM and VLAM on neovascularization in wounds was determined by alpha smooth muscle actin (αSMA) (a) and CD31 (b) staining. White arrows (a & b, middle and right panels) indicate areas with new blood vessels in VCAM- and VLAM-treated wounds. (c) The formation of the basement membrane in control (saline gel), VCAM- and VLAM-treated wounds was detected by collagen IV (Col IV) staining. White asterisks highlight the basement membrane. A saline gel-treated group served as the negative control. A total of four animals was used for each group. Samples were co-stained with DAPI (blue) to visualize cell nuclei.

Restoration of blood flow in wounds is required for healing and is achieved through neovascularization, or the formation of new blood vessels. To examine the effects of VLAM and VCAM on neovascularization, we evaluated wound tissue sections for the presence of blood vessels using blood vessel-specific markers. Samples were stained with fluorescent-labeled antibodies for alpha smooth muscle actin (αSMA), a marker of smooth muscle cells within blood vessels (Fig 4A), and platelet endothelial cell adhesion molecule, also known as CD31, an endothelial cell marker (Fig 4B). Tissues were co-stained with DAPI (blue) to visualize cell nuclei. As shown in Fig 4, both αSMA and CD31 were significantly increased in VCAM- and VLAM-treated wounds (Fig 4A and 4B, middle and right panels) as compared to control wounds (Fig 4A and 4B, left panels*)*. The white arrows indicate αSMA- (Fig 4A, middle and right panels) and CD31- (Fig 4B, middle and right panels) positive blood vessels, demonstrating that more blood vessels were formed in wounds treated with VCAM and VLAM than in control wounds (Fig 4A and 4B).

The basement membrane in skin represents the dermal-epidermal interface that anchors the epidermis and dermis and is essential for the proper assembly of skin layers [43]. This membrane was visualized by collagen IV staining of control, VCAM- and VLAM-treated wound tissue (Fig 4C). White arrowheads highlight the basement membrane in VCAM-and VLAM-treated wounds (Fig 4C, middle and right panels). The basement membrane was not formed in control wounds (Fig 4C, left panel, white asterisks highlight the basement membrane*)*.

## Discussion

VCAM described in this study is a commercial placental product that is used for management of acute and chronic wounds and for a variety of surgical procedures [21,24,44]. VCAM retains all native components of fresh AM including viable cells and a full spectrum of biological activities associated with fresh tissue [17,20,45]. Our VCAM cryopreservation method includes a DMSO- and HSA-based solution that results in high cell viability within the preserved tissue [17,18]. Previous studies on devitalized VCAM have demonstrated that viable cells are required for the tissue to respond *in vitro* in a wound-mimicking environment [18,45,46]. Clinical studies have demonstrated that VCAM results in a higher rate of wound closure in comparison to devitalized amnion-chorion for intractable large or sinus track wounds [26,27].

In this study, using VCAM as the positive control, we evaluated the effect of VLAM in a chronic wound diabetic mouse model. Our *in vivo* results show that complete wound closure was achieved in both VCAM- and VLAM-treated groups of animals but not in the control (saline gel treated) group. Importantly, histological analysis of samples revealed restoration of normal skin structure after wound closure in VCAM- and VLAM-treatment groups. Three pieces of data support this conclusion. Firstly, the presence of “basket weave”-like structures indicates that collagen deposited in the wound underwent maturation in VCAM- and VLAM-treated samples (Fig 3C). Secondly, wounds were completely vascularized in both VCAM and VLAM samples compared to the control, as demonstrated by new blood vessel formation (Fig 4A & 4B). Finally, a well-established basement membrane was observed in both VCAM- and VLAM-treated wounds but not in the control (Fig 4C). While activities of devitalized cryopreserved or lyopreserved AMs were not assessed in the present study, biological activities of VCAM and devitalized cryopreserved AM have been previously reported [17,18,20,45]. These studies found that devitalization of AM decreases wound-relevant anti-inflammatory, antioxidant, antimicrobial, and angiogenic activities of the membrane [17,18,20,45]. Taken together, these data suggest that VLAM and VCAM are comparable in the ability to support the healing of chronic wounds and that preservation of all components of AM, including viable cells, is essential.

Published reports support the safety and effectiveness of DMSO in preserving viable cells within amniotic tissue for clinical use [17,18,21,24,26]. In dermatological applications, topical use of DMSO has been shown to be both safe and beneficial [47]. VCAM retrospective and prospective clinical studies reported positive clinical outcomes in patients with acute and chronic wounds of various etiologies and in a variety of surgical procedures without adverse events attributed to VCAM application [22–25]. Data from these VCAM clinical studies are consistent with other reports describing the clinic use of DMSO-cryopreserved viable AM prepared using different cryopreservation protocols [48,49]. In a randomized controlled clinical trial, 75% of pressure ulcers were closed in the cryopreserved AM group versus 0% in the control group [48]. A different retrospective study reported positive outcomes with DMSO-cryopreserved viable AM for a broad variety of ocular diseases and dermatological defects without adverse events [49].

Glycerol, another preservative agent, is routinely used for amniotic membrane preservation. Studies have shown that glycerol is cytotoxic, therefore it does not preserve viable amniotic tissue [50]. In certain applications, when there are no reported differences in outcomes for viable and non-viable amnion the use of non-viable tissue conveniently stored at ambient temperatures is preferred. For example, previous studies have reported the use of non-viable cryopreserved or dehydrated amniotic membrane for ophthalmologic surgeries as well as for reconstructive urology procedures [50,51]. In addition, non-viable AM is used as a scaffold for cell culturing. In particular, limbal epithelial cells cultured with denuded AM grow more efficiently than those cultured with cryopreserved AM [50]. Although denuded, devitalized, and decellularized amniotic membranes are broadly used for different clinical applications, growing scientific and clinical evidence demonstrate the importance of preserving all components of amniotic membrane, including endogenous viable cells for retention of the full spectrum of AM functional properties [17,18,20,24,26,46,52,53].

A caveat of cryopreservation is that it requires ultra-low temperature storage and shipment equipment that limits widespread use of VCAM and other therapies containing living cells. The complex logistics for storage and distribution of viable cells and tissues is a challenge that plays a significant role in delaying progress in the field of cell therapy. Viable cells have a short shelf-life at ambient temperatures that complicates their storage and delivery to patients. Development of a preservation technology for long-term ambient storage of living tissues would have tremendous practical significance.

Lyophilization, or freeze-drying, is a form of desiccation that involves the sublimation of water from a frozen sample and has been widely used in stabilizing pharmaceuticals and biologics [54]. Lyophilization is also a common method of preserving tissue including AM, but viable cells in the tissue are compromised during lyophilization resulting in non-viable tissues. For desiccation-tolerant cells like bacteria and yeast, lyophilization is the current method of choice for long-term ambient storage without loss of cell viability [55,56]. Desiccation-tolerant cells have high concentrations of small carbohydrates (di- and trisaccharides), such as sucrose, maltose, trehalose or raffinose, that protect intracellular molecules and organelles from damage [35]. A better understanding of the protective mechanisms of desiccation-tolerant cells provided the fundamental basis to develop lyophilization methods for desiccation-sensitive cells. Multiple cell-preservation agents, including other sugars, proteins, polymers, lipids, and amino acids have been identified [31,57–59] and used in the dehydration of a broad variety of primary cells and cell lines [60–62]. The presence of viable cells after rehydration of hematopoietic and mesenchymal stem cells [36] and functional red blood cells and platelets [63], has been reported. While “proof of concept” data exists, a number of issues remain to be addressed. These issues include reduced cell viability and functionality, poor reproducibility, difficulty with upscaling, and long-term storage time. For example, oxidation of hemoglobin during dehydration triggers hemolysis that prevents effective preservation of red blood cells [64]. Zhang and colleagues reported up to 70% cell viability after rehydration of lyophilized mesenchymal stem cells, but these cells did not proliferate [37]. Successful nuclear transfer was achieved with lyophilized sheep granulosa cells after 3 years of dry storage, but viability of those cells was poor [33]. Natan *et al*. developed a lyophilization protocol involving directional freezing in a solution containing both trehalose and an antioxidant [36]. This method resulted in viable and functional cord blood hematopoietic stem cells, however, testing was performed after only one week of dry storage. Recent studies have shown that lyophilization preserves DNA and RNA integrity in cells and tissues [34]. However, following lyophilization, cell viability was compromised in one study [34], and tissue cell viability was not investigated in the other study [65].

Based on accumulated data on cell preservative agents, lyophilization processes, and preliminary literature protocols for mammalian cell drying, we developed a lyopreservation technique resulting in living tissues that could be stored at ambient temperatures [66]. In the present study, we processed fresh AM using this method, evaluated the structural and functional properties of VLAM and compared those to fresh AM and VCAM. Analyses confirmed that tissue architecture (Fig 1C), growth factors (Fig 1D), and viable cells (Fig 1E) of fresh AM are preserved in both VCAM and VLAM after six months storage, the longest time point tissues were evaluated in this study.

The challenges with evaluation of tissue cell viability are its thickness, the presence of different structural layers, and inherent variability in cell distribution within the tissue. Cell viability in this study was assessed by direct staining of AM with fluorescent dyes specific for living and dead cells. To overcome inherent cell distribution and viability within the tissue we assessed cell viability using randomly taken microscopic field images of many different areas of the tissue sample from 50 donors. There were no statistically significant differences in cell distribution and viability between fresh AM, VCAM and VLAM. *In vitro* and *in vivo* experiments demonstrated that both VLAM and VCAM retain anti-inflammatory, and proangiogenic properties of AM that correlated with faster closure of wounds in diabetic mice (Figs 2–4). Proliferation and differentiation of VLAM cells were not investigated, however, results show that VLAM is comparable to VCAM in wound-relevant *in vitro* and *in vivo* functional models. Based on these data we believe that VLAM will provide the same clinical benefits as VCAM in the management of acute and chronic wounds [21]. Importantly, VLAM offers a greater advantage over VCAM in that it can be stored at ambient temperatures.

A number of studies reporting tissue lyophilization in the presence of lyoprotective agents have been focused on evaluation of tissue architecture and growth factors. Of these, only a few studies have described the effects of freeze-drying on cell viability in tissue-like constructs. It has been shown that the presence of trehalose during freeze-drying can help preserve native AM morphology better than in its absence [67] and better than cryopreservation [68]. A cryodesiccation method using trehalose and/or DMSO for preservation of tissue-engineered skin substitutes (TESs) containing fibroblasts has been reported [69]. Fibroblast viability in freeze-dried TESs was less than 40% of the fresh control TES after one week of storage. However, even with reduced cell viability, TESs provided more benefit to mouse wounds in comparison to an acellular construct [69].

This study is the first demonstration that lyophilization of trehalose-saturated AM results in viable tissue after six months of storage at room temperature. Evaluation of cell viability in lyopreserved tissues after long-term storage is in progress. The results presented in this study indicate that the new method for preservation and ambient storage of AM: i) is reproducible and ii) provides extended tissue shelf life. The benefits of this optimized lyopreservation technology may accelerate the development, commercialization, and widespread use of products containing living cells. The molecular mechanisms of living tissue lyophilization and applicability for other types of tissues including vascularized tissues and organs remained be addressed in future studies. The benefits of a lyopreservation technology allowing for ambient storage of living cells and tissues may accelerate the development, commercialization, and widespread use of products containing living cells.

## Supporting information

S1 Fig Effects of VCAM and VLAM over a 35-day period in a diabetic mouse chronic wound model. **(a)** Representative images of wounds (n = 6) taken at one-week intervals. **(b)** Animal weight measured weekly and expressed as a percent of baseline weight at day 0. The saline gel-treated group served as a negative control.
(TIF)
