# Supplementary figures and images for: Properties of viable lyopreserved amnion are equivalent to viable cryopreserved amnion with the convenience of ambient storage
Source: PLoS One. 2018 Oct 2;13(10):e0204060. doi: 10.1371/journal.pone.0204060 (PMC6168127; doi:10.1371/journal.pone.0204060)

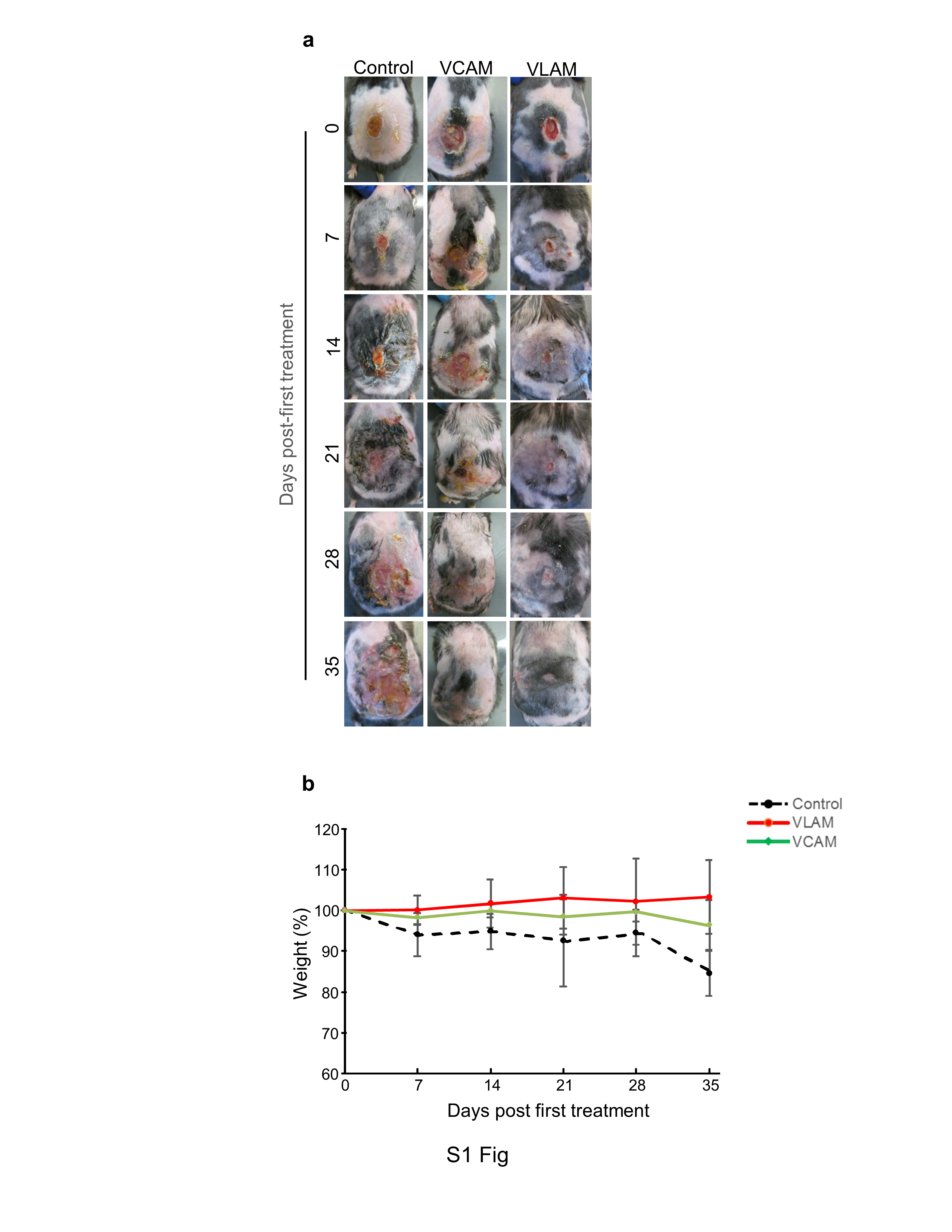

Supplement: S1 Fig — (a) Representative images of wounds (n = 6) taken at one-week intervals. (b) Animal weight measured weekly and expressed as a percent of baseline weight at day 0. The saline gel-treated group served as a negative control. (TIF) [file pone.0204060.s001.tif]
